# Reliable estimation of internal oscillator properties from a novel, fast-paced tapping paradigm

**DOI:** 10.1038/s41598-022-24453-6

**Published:** 2022-11-28

**Authors:** Ece Kaya, Molly J. Henry

**Affiliations:** 1 Max Planck Institute for Empirical Aesthetics, Frankfurt, Germany; 2 Toronto Metropolitan University, Toronto, Canada

**Keywords:** Human behaviour, Perception, Attention

## Abstract

Rhythmic structure in speech, music, and other auditory signals helps us track, anticipate, and understand the sounds in our environment. The dynamic attending framework proposes that biological systems possess internal rhythms, generated via oscillatory mechanisms, that synchronize with (entrain to) rhythms in the external world. Here, we focused on two properties of internal oscillators: *preferred rate*, the default rate of an oscillator in the absence of any input, and *flexibility*, the oscillator’s ability to adapt to changes in external rhythmic context. We aimed to develop methods that can reliably estimate preferred rate and flexibility on an individual basis. The experiment was a synchronization—continuation finger tapping paradigm with a unique design: the stimulus rates were finely sampled over a wide range of rates and were presented only once. Individuals tapped their finger to 5-event isochronous stimulus sequences and continued the rhythm at the same pace. Preferred rate was estimated by assessing the best-performance conditions where the difference between the stimulus rate and continuation tapping rate (tempo-matching error) was minimum. The results revealed harmonically related, multiple preferred rates for each individual. We maximized the differences in stimulus rate between consecutive trials to challenge individuals’ flexibility, which was then estimated by how much tempo-matching errors in synchronization tapping increase with this manipulation. Both measures showed test–retest reliability. The findings demonstrate the influence of properties of the auditory context on rhythmic entrainment, and have implications for development of methods that can improve attentional synchronization and hearing.

## Introduction

Adapting to and coping with the dynamically changing environment around us requires continuous tracking of sensory input, extraction of relevant information, and anticipation of upcoming events. These processes are critical in everyday experiences such as appreciating a complex musical piece or simply holding a conversation in a noisy restaurant. In the auditory domain especially, dealing with temporal information from the environment hinges on the ability to adapt and adjust our attention on a moment-by-moment basis. Here, we work from the observation that the inherent rhythmic structure of sounds drives our attention in a time-varying manner, and aim to understand individual differences in our abilities to track temporal structure in a fast-paced environment.

The literature on how we perceive time and rhythm is clustered around two main perspectives: interval and oscillator models. According to interval models, time perception is governed by an “internal clock” where a pacemaker generates periodic pulses and elapsed time is represented by their count^1^. Oscillator models, perhaps most notably, the dynamic attending framework, propose that biological systems possess internal rhythms, generated via oscillatory mechanisms, that synchronize with rhythms in the external world by adapting their phase and period—this process is referred to as *entrainment*^2^. The natural frequency, i.e., *eigenfrequency*, of an internal oscillator is fixed at a rate that is specific to that oscillator, regardless of the properties of the sensory input the oscillator is responding to ^3^. One critical prediction of this account is that stronger coupling is possible between oscillators with similar natural frequencies, i.e., smaller *detuning*^4^, than those with dissimilar frequencies, since the latter would require more adaptation. Synchronization between the organism’s own internal rhythm and an external stimulus rhythm is successful to the extent that the oscillator’s natural frequency matches the stimulus rate, which leads to a restricted range of rates around the organism’s preferred rate within which it can accomplish 1:1 synchronization^5^.

Here, we adopted the dynamic attending perspective and focused on two properties of internal oscillators that we assume to underlie perception and production of rhythm: their intrinsic, natural frequencies, referred to here as *preferred rate*; and their *flexibility*, the extent to which they are able to adapt to changes in the external rhythmic context. One common behavioral measure of preferred rate is ‘spontaneous motor tempo’ (SMT), the tempo at which individuals tap their finger^6–8^ or a drumstick^3^ on a desk or a sensor when asked to move at a “comfortable rate”. SMT (mean or median of the intervals between the individual taps) generally clusters around 500–600 ms for adults^6,7,9^, but varies fairly widely from person to person. In addition, studies report slowing of tapping rate with age^3,6^ and musical experience in children^3^, as well as variation over the circadian cycle^9^. SMT is also correlated with the preferred perceptual tempo, suggesting that same underlying mechanisms might be shared by both perception and production of rhythm (preferred period hypothesis) (Ref.^6^, but see Ref.^10^).

SMT appears to act as an ‘anchor’ during tapping tasks where stimulus rate varies. During synchronization-continuation tapping tasks, participants first synchronize their taps with a metronomic stimulus, and then are tasked with continuing to tap at that same rate after the stimulus ceases. However, during continuation of a rhythmic stimulus, individuals tend to drift back towards their SMT^11,12^ and over- and underproduce stimuli that are faster and slower than their SMT, respectively^4,6,7,12^.

Despite a focus on the natural frequency of internal oscillators in timing literature, not much attention has been given to their other primary property: flexibility (referred to as elasticity in Ref.^13^ and adaptability, the compliment of stability, in Ref.^14^). In the current study, we defined flexibility as the ability of an internal oscillator to adapt to changes in external rhythmic stimuli: here in particular we focus on period adaptation in response to changes in stimulus rate across trials. The consequences of flexibility should be visible in the magnitude of so-called history effects. In the context of rhythm production, tapping responses generated by a fully flexible oscillator would be devoid of effects of stimulus history because the oscillator would adapt to each new stimulus rate fully and easily. However, an inflexible oscillator will have trouble adapting to a new rate, and tapping responses will reflect the rhythmic properties of the preceding trials. The effect of immediate context on stimulus perception is referred to as serial dependence^15–17^ or hysteresis^18^. In the context of the dynamical systems approach to human behavior, hysteresis refers to the system’s tendency to remain in a certain state, even after a parameter changes direction^19^. After the change (stimulus rate here), the system continues to oscillate at the previously-entrained rate. As consequence, the time the oscillator takes to entrain to the new stimulus as well as the strength of entrainment depend on the parameters responsible for adaptation, which differ between different oscillator models (phase and period correction in Ref.^20^, coupling strength and learning rate in Ref.^21^, coupling strength and period adaptation rate in Ref.^2^). Thus, the extent to which tapping responses would be affected by the stimulus history depends on the adaptation ability of the underlying oscillatory mechanisms.

Individuals’ ability to adapt to changes in temporal context is often investigated by ‘perturbation paradigms’^22–24^ where either the onset times of individual events (phase) or the duration between consecutive events (period) in an otherwise isochronous stimulus sequence is manipulated. Key findings of this work are faster adaptation to phase than period perturbations^22,23^ and an asymmetry between adaptation to sequences that slow down and those that speed up, with the former resulting in smaller asynchronies of taps relative to stimulus events^22–24^. Though perturbation studies provide insight into individuals’ abilities to adapt and strategies for adaptation, they examine effects of temporal changes of tapping responses in short time windows local to the perturbation. In the current study, we focus on the effects of stimulus history in a broader context that extends to a preceding trial in a multi-rate session. To our knowledge, only one study has examined serial dependence in production of rhythmic stimuli with different rates that were presented in different trials. In that study, rate reproductions were positively influenced by the rate of the stimulus on the immediately preceding trial^17^.

Interval and oscillator models of rhythmic synchronization make similar predictions regarding adaptation to changes in stimulus rate: both models assume continuous adjustment of the internal period (or timekeeper) in response to variation in stimulus intervals. A critical distinction between the models is that oscillator models predict an asymmetry between responses to a speeding-up vs. a slowing-down rate change whereas interval models predict no difference. The asymmetry stems from the sinusoidal period adaptation function in the oscillator model^23^. The predictions of oscillator models have been confirmed by the finding that synchronization to a slowing-down stimulus results in smaller asynchronies between stimulus onsets and taps than to a speeding-up stimulus^22–24^. Moreover, comparisons between interval and oscillator models in time judgments^25^ and sensorimotor synchronization^24^ showed that oscillator models were better at explaining behavioral results and accounting for temporal adaptation.

Although there is evidence for individual differences in proneness to history effects^15,26^, these findings have generally been reported as secondary results of the studies and were not their main focus. Moreover, studies revealing age- and experience-related differences in preferred rate^3,6^ typically estimate preferred rate by means of a single task, SMT, which has been shown to be influenced by dynamical factors such as arousal and circadian cycle^9^. Finally, in paradigms where individuals’ rate preferences^3,6^ or tapping performance^6,7,27^ is compared across stimulus-rate conditions, those conditions are typically low in number, unrelated to SMT, and poorly resolved, prohibiting a precise comparison to preferred rate as measured by SMT.

The aims of the current study were to explore the preferred rate and flexibility properties of internal oscillators and develop methods for their estimation on an individual basis. By characterizing individual differences in oscillator properties and their relation to rhythmic entrainment, we aim to gain understanding about individuals’ synchronization patterns to the external stimuli in real world context (i. e., situations that improve or disrupt rhythmic entrainment), which in turn may help us develop methods to optimize individuals’ synchronization and hearing by fine-tuning parameters of sound or stimulation^28^.

**Figure 1:**
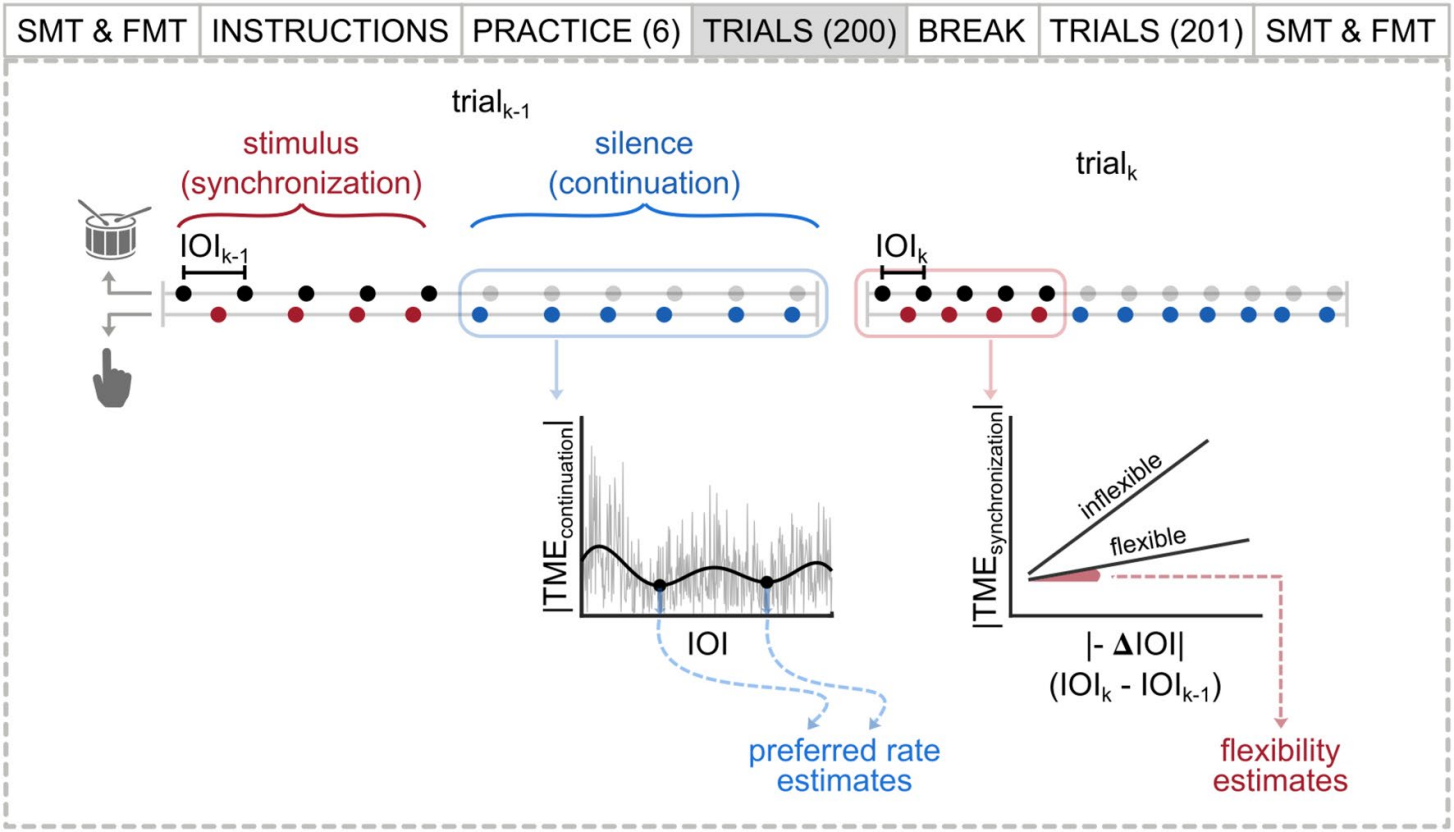
The experiment procedure of a single session, and illustration of the methods used to estimate preferred rate and flexibility. The horizontal lines represent the temporal structure of events in two consecutive trials. Dots on the top line represent the isochronous stimulus; audible sounds in black, and inaudible, theoretical timestamps in transparent gray. Dots on the bottom line represent synchronization (red) and continuation (blue) taps. Tempo-matching errors (TME) were calculated as the difference between stimulus IOI and tapping rate, separately from synchronization and continuation sections of the trials. Individuals’ preferred rates were estimated by fitting curves to their |TME_continuation_| values over the IOI range and obtaining their local minima. Flexibility was estimated by fitting linear models to individuals’ single-session data where |TME_synchronization_| was predicted by |− ΔIOI|, and obtaining slopes, with a smaller value indicating more flexibility.

In the current study, we introduced a novel measure for preferred rate in addition to the SMT task we included in our design. The experiment was a synchronize-continue tapping paradigm where we asked participants to synchronize their taps with an isochronous stimulus sequence and continue the rhythm at the same rate. We used a wide and finely sampled range of stimulus inter-onset intervals (IOIs) that enabled high-resolution preferred rate estimation. In order to challenge oscillator flexibility, we introduced conditions where IOI differences between consecutive trials (ΔIOI) were maximized. Across all trials within the experiment, the distribution of ΔIOI was balanced between large and small changes, as well as negative and positive changes (whether a trial was faster or slower than the previous), which enabled systematic comparisons of tapping performance for quantification of oscillator flexibility. The experiment procedure and the illustration of the methods used to estimate individuals’ preferred rate and flexibility are presented in Fig. 1.

### Hypotheses

Our first hypothesis was that tempo-matching errors calculated across both the synchronization and continuation sections of the trial (TME_all_) would have a negative slope as a function of stimulus rate (IOI), arising from fast stimuli being tapped too slow and slow stimuli being tapped too fast. The dynamical systems approach predicts reduced asynchronies between stimulus and the oscillator as the difference between the stimulus frequency and the oscillator’s natural frequency (i.e., detuning) is reduced^13^ since it would require more energy to coordinate motion at other frequencies^12^. In addition, since finger tapping is rate-limited due to motor and cognitive constraints^21^, and since we anticipated that most participants’ preferred rates would fall somewhere into the middle of the range, we expected tapping errors to increase at both ends of the IOI range.

The second hypothesis was that tempo-matching errors during continuation tapping (TME_continuation_) would be maximized when stimulus rate was far from the individual’s preferred rate, based on the findings of drift towards preferred rate^11,12^, reduced tapping errors at one’s SMT^13^, and over-and-underproduction of rates away from preferred rate^4,6,7,12^.

Finally, we hypothesized that larger trial-to-trial rate changes would result in increased tempo-matching errors during synchronization. During re-synchronization to a new stimulus, the oscillator updates its phase and period in response to each interval^20^, which would result in a gradually decreasing discrepancy between the phase and period of the stimulus and the oscillator assumed to underly the tapping responses. We predicted hysteresis due to the large changes in stimulus rate across trials (ΔIOI) which would result in the oscillator adapting gradually to the upcoming stimulus rate, leading to tempo-matching errors during synchronization (TME_synchronization_). Studies on temporal adaptation reveal asymmetries between synchronization to a speeding-up stimulus and slowing-down stimulus with the former resulting in smaller asynchronies^22–24^, as predicted by oscillator models^23^. Thus, regarding synchronization tapping, we also hypothesized that the effect of rate change on tempo-matching errors would be the stronger in cases where the new rate is faster than previous.

## Results

In this section, we first present findings for the overall tapping patterns across the full range of stimulus rates. Then, we present estimates of preferred rate and oscillator flexibility, followed by a descriptive analysis of the unpaced tapping task. Finally, we report results for the comparisons of our measures across sessions and tasks.

### General tapping patterns

**Figure 2:**
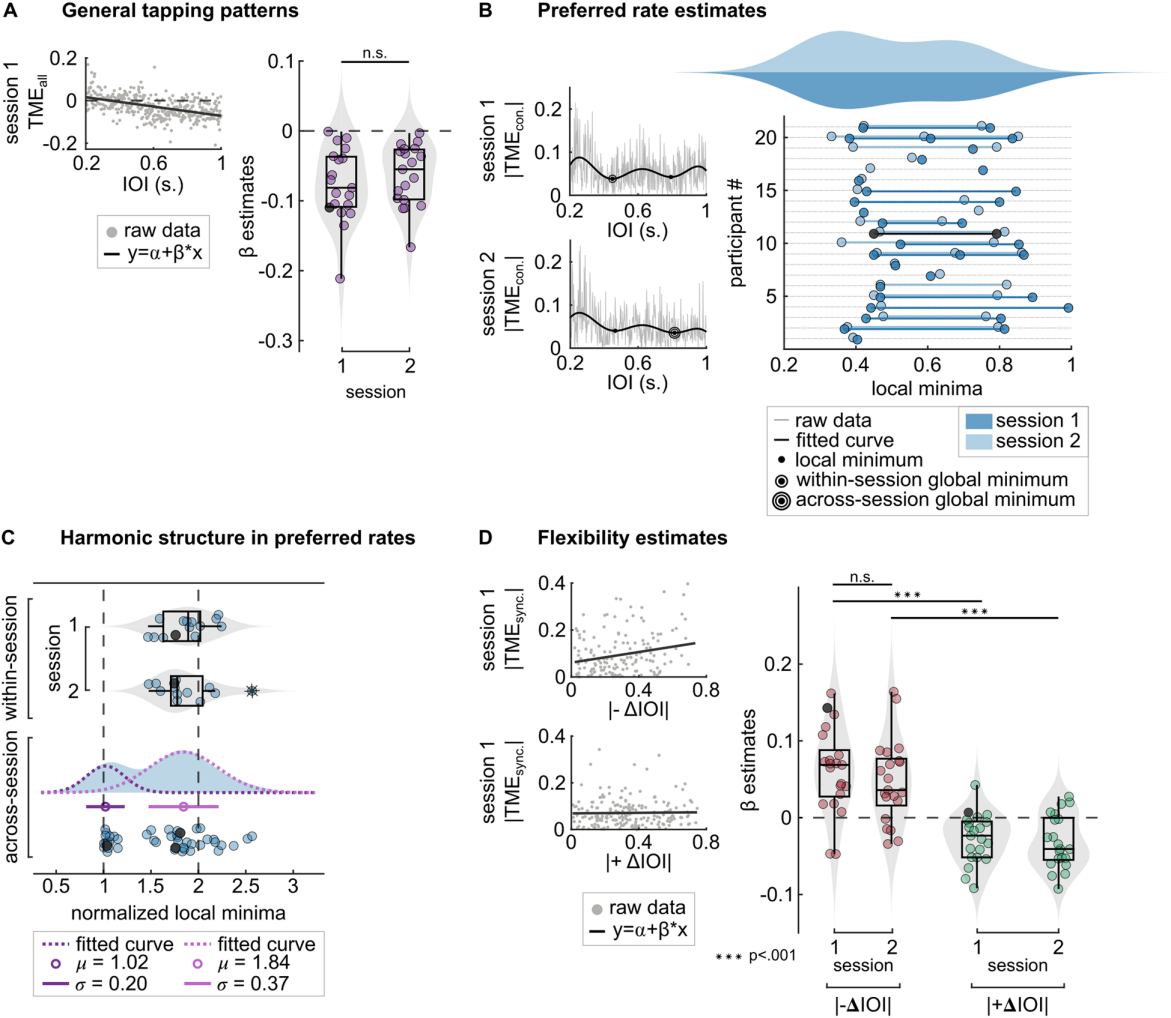
Main findings. (**A**) General tapping patterns across the range of stimulus IOIs. Left: an example participant’s session 1 raw data and the fitted least-squares line. Right: β estimates (slopes) obtained from the linear models (TME_all_ = α + β × IOI), in individual sessions. Box plots show median (black horizontal line), 25th and 75th percentiles (box edges) and extreme datapoints (whiskers). Each circle represents a single participant. Black circle represents β of the line in the example fit on the left. (**B**) Preferred rate estimates. Left: an example participant’s raw data and the fitted curves in session 1 (top) and session 2 (bottom). Individuals’ preferred rates were the stimulus IOI at the local minima of the fitted curves, where the predicted |TME_continuation_| was minimum. Among the local minima, the ones with less predicted TME were the global minimum, obtained within and across sessions. Right: 21 participants’ local minimum estimates and the resulting distributions in session 1 (dark) and session 2 (light). Each circle represents a local minimum and the black circles represent the example participant’s estimates. (**C**) Harmonic structure in preferred rates. The plot shows normalized local minima, where each individual’s estimate was divided by the smallest value obtained within session 1 and 2 (top and middle, respectively) and across sessions (bottom). Dotted curves represent the gaussian curves, fitted to the kernel density of the bimodally distributed data. Single circles and the same-color whiskers represent mean and standard deviation of the fitted theoretical distributions. Each circle represents a normalized local minimum and the black circles represent the example participant’s estimates. Box plots show median (black vertical line), 25th and 75th percentiles (box edges) and extreme datapoints (whiskers). (**D**) Flexibility estimates. Left: an example participant’s session 1 data and the linear fits where |TME_synchronization_| was predicted by absolute negative ΔIOI (top) and by positive ΔIOI (bottom). Right: β estimates (slopes) obtained from the linear models where |TME_synchronization_| was predicted by absolute negative ΔIOI (red) and by positive ΔIOI (green), in individual sessions. Each circle represents a single participant. Black circles represent β of the lines in the example fit on the left.

In order to assess how tapping performance depended on stimulus rate, we first explored the patterns of tempo-matching error, TME, across participants. To do so, we fitted linear regression models to each individual’s single-session data, where TME_all_ was predicted by stimulus IOI. Figure 2A shows the least-squares fit for an example participant’s data and the distribution of the resulting estimated linear coefficients (β) for session 1. Single-sample t-tests confirmed that that β estimates were significantly smaller than zero in session 1 (M = − 0.077, SD = 0.052; t(18) = − 6.4105, p < 0.001) and in session 2 (M =  − 0.071, SD = 0.053; t(19) =  − 5.9719, p < 0.001). See “Comparisons between sessions” section.

Based on an entrainment approach, we predicted that tempo matching would be most accurate near each individual’s preferred rate. In line with this prediction, we observed that most participants tapped too fast at slow rates and too slow at fast rates, indicating the existence of a range of rates where participants matched stimulus IOI most accurately. Our next step was to quantify this range.

### Preferred rate estimates

We quantified the stimulus IOIs where tempo-matching error during continuation tapping was minimal by fitting sum-of-sine functions to |TME_continuation_| values separately for each individual in each session and obtaining the local minima of the fitted curves (see “Methods” section). For each participant, we estimated up to 3 local minima as an index of preferred rate(s). The distribution of local minima across both sessions is presented in Fig. 2B. For both sessions, local minima across all participants showed a bimodal distribution with peaks around 400 ms and 800 ms.

In order to quantify the bimodality of the observed distribution, for each individual, we divided all fits' local minima by the smallest local minimum, but then excluded the denominators (the smallest local minimum) from the final distribution, as they were always equal to 1. We performed this normalization procedure first for participants’ single-session local minima estimates, and then for combined estimates from both sessions. The resulting distributions for first and second sessions are presented in Fig. 2C. A single point of normalized local minimum = 2.57 (marked in Fig. 2C, middle) exceeded 3 × the median absolute deviation (MAD) of all normalized local minima in session 2, and was detected as an outlier. If local minima were harmonically related, we would expect values centered on 2 after normalization. Separate single-sample t-tests showed that the mean normalized local minima from session 1 (M = 1.869, SD = 0.251) and session 2 (with the outlier: M = 1.862, SD = 0.296; without the outlier: M = 1.804, SD = 0.216) did not differ from 2 (session 1: t(13) =  − 0.5029, p = 0.623; session 2: t(12) =  − 0.4486, p = 0.662 with, t(11) =  − 0.8745, p = 0.401 without the outlier). Moreover, we repeated this same normalization analysis for data pooled across sessions, and fitted gaussian probability density functions to the resulting bimodal distribution. Normalized local minimum estimates from pooled session data, presented in Fig. 2C, showed a bimodal distribution with the estimated parameters of μ_1_ = 1.02, σ_1_ = 0.2 and μ_2_ = 1.84, σ_2_ = 0.37, suggesting test–retest reliability and consistency in harmonic structure of local minima estimates across sessions.

**Figure 3:**
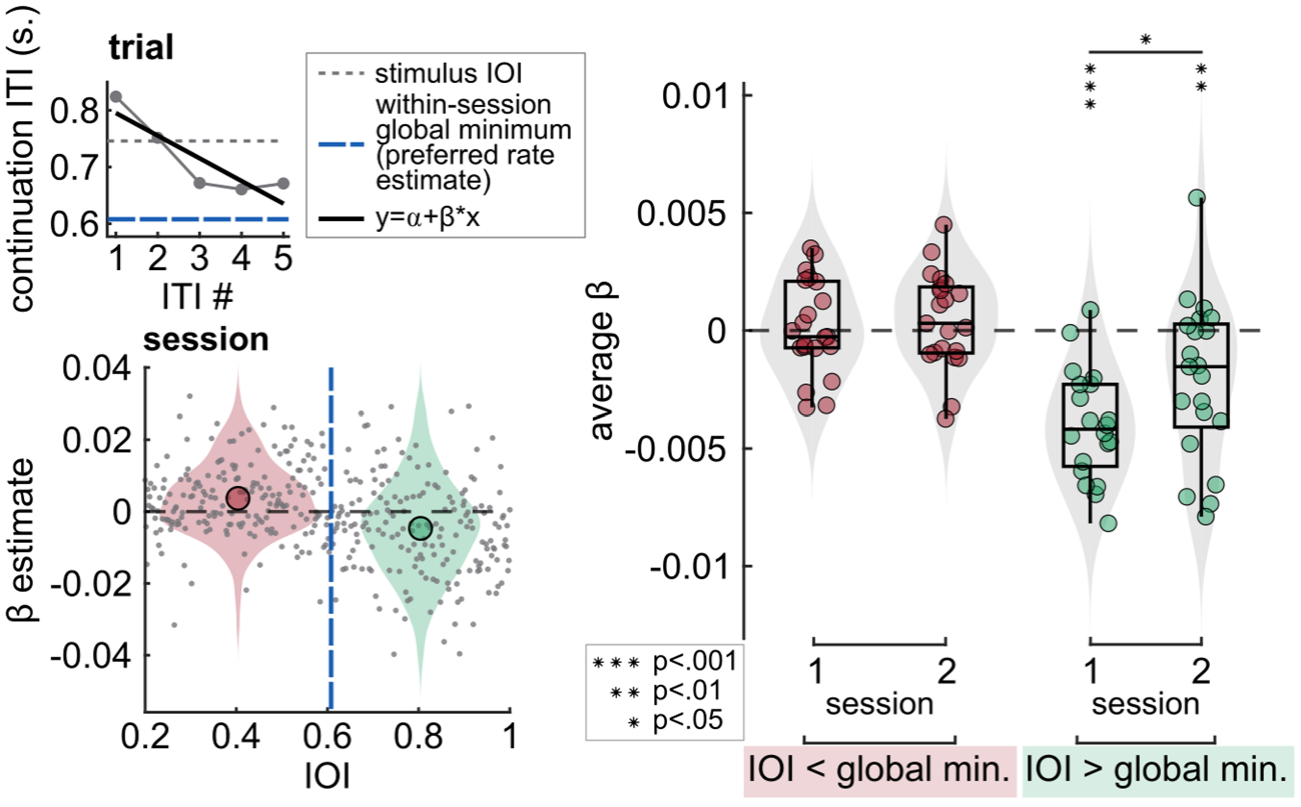
Results of the drift analysis. Left, top: Linear fits to an example trial’s tapping data. Connected dots represent the continuation ITIs, dashed lines are stimulus IOI (grey), and participant’s within-session global minimum estimate, i.e., preferred rate (blue). Drift on individual trials was quantified as the slopes (β) of the fitted least-squares lines (black). Left, bottom: β estimates obtained from an example participant’s single-session data. Gray dots represent β from individual trials. The dashed blue line represents the participant’s preferred rate estimate (within-session global minimum). Circle on the left (red) is the mean of β values obtained from the trials where IOI was faster than the preferred rate estimate, circle on the right (green) is the mean of β values obtained from the slower-than-preferred rate trials. Right: Distribution of β estimates, averaged within individual sessions. Each circle represents an individual’s average β. Average β from trials where stimulus IOI was faster than the individual’s preferred rate estimate are marked in red, average β from trials where stimulus IOI was slower than the individual’s preferred rate estimate are marked in green. Box plots show median (black vertical line), 25th and 75th percentiles (box edges) and extreme datapoints (whiskers).

We expected participants to drift back towards their own preferred rates once the stimulus ceased, i.e., during continuation. We therefore investigated whether participants drifted towards their preferred rate during continuation tapping or whether they simply overproduced fast and underproduced slow rates without drifting gradually away from them. We did this by assessing the change in the tapped interval duration (intertap interval, ITI) over the course of each trial’s continuation section. Slopes obtained from linear models (ITI = α + β × tap number) showed the direction of the change: positive slopes indicated a slowing of the tapping rate and negative slopes indicated that the tapping during continuation got faster. If individuals drifted towards their preferred rate, the slopes obtained from trials with stimuli faster than the preferred rate should be positive, and slopes obtained from trials with slower stimuli should be negative. Results showed significant deviation from zero only for slopes from slower-than-preferred rate trials (session 1: M =  − 0.004, SD = 0.002; t(19) = − 7.723, p < 0.001; session 2: M =  − 0.002, SD = 0.003; t(20) = − 2.823, p = 0.005). Slopes from faster-than-preferred rate trials did not differ from zero. Interestingly, slopes from slower-than-preferred rate trials in session 2 were significantly higher than those in session 1 (t(19) =  − 2.394, p = 0.027). Drift analysis for an example participant’s data and the resulting distributions of average slopes are shown in Fig. 3.

In summary, we present a method that estimates preferred rate by quantifying the stimulus rates where an individual most accurately maintained the stimulus rate during continuation tapping, that is, where their tapping rate did not drift away from the stimulus IOI after the stimulus was removed. The estimates were in line with the predictions of the preferred period hypothesis and entrainment account, as we were able to identify rates with minimal tapping errors, which were also harmonically related to each other.

### Flexibility estimates

As a first step to quantify the effect of ΔIOI on tempo-matching errors during synchronization to estimate flexibility, we fitted linear models to individual-participant, single-session data. We fitted separate models to participants' single-session data where |TME_synchronization_ | was predicted either by |− ΔIOI| or |+ ΔIOI|. All β estimates are presented in Fig. 2D. Separate one sample t-tests showed that, β estimates for |− ΔIOI| were significantly larger than zero both in first (M = 0.061, SD = 0.055) and second (M = 0.047, SD = 0.053) session (session 1: t(20) = 5.0511, p < 0.001; session 2: t(20) = 4.0405, p = 0.001). In contrast, β estimates for |+ ΔIOI| were significantly smaller than zero both in first (M =  − 0.027, SD = 0.032) and second (M =  − 0.031, SD = 0.035) session (session 1: t(20) =  − 3.848, p = 0.001; session 2: t(20) =  − 4.155, p < 0.001). Paired-samples t-tests revealed that β estimates from the equation |TME_synchronization_|= α + β ×|− ΔIOI| were also larger than β estimates from the equation |TME_synchronization_|= α + β ×|+ ΔIOI| in both session 1 (t(20) = 7.783, p < 0.001) and session 2 (t(20) = 5.556, p < 0.001). Together, these results suggest that whereas |− ΔIOI| increased tempo-matching errors during synchronization, |+ ΔIOI| decreased them.

In summary, we assessed individuals’ adaptation to rapid rate changes as a means to quantify their oscillator flexibility. For this analysis, we focused on synchronization tapping and predicted that the most challenging task would be synchronizing to stimuli that were faster than previous. To explore the effects of ΔIOI on TME_synchronization_, we fitted linear models to each set of conditions where absolute TME_synchronization_ was predicted by either positive or negative ΔIOI. In both sessions, β estimates obtained from these fits showed that |− ΔIOI| inflated TME_synchronization_ whereas |+ ΔIOI| affected TME_synchronization_ in the opposite direction, and thereby poses no challenge to individuals’ adaptation and oscillator flexibility. Our final estimates for oscillator flexibility thus consisted of β estimates obtained from this most challenging condition where individuals synchronized to faster-than-previous stimuli.

### Unpaced tapping

In a single experimental session, each participant completed a series of unpaced tasks, where they tapped their finger on the desk without hearing any sounds. In the spontaneous motor tempo (SMT) task, they tapped at a comfortable rate, and in forced motor tempo (FMT) tasks, they first tapped at the slowest, then at the fastest rate that was comfortable to maintain. Participants’ unpaced tapping rates from session 1 are presented in Supplementary Fig. S2. For each task, paired-samples t-tests quantified the difference in rates before and after the session. SMT rates after the experimental session (M = 0.580, SD = 0.207) were more variable than those from before the session (M = 0.555, SD = 0.160) but the rates did not significantly differ (p = 0.172) and were significantly correlated (r(19) = 0.711, p = 0.001). The ‘fastest’ FMT rates clustered around 200 ms, did not differ before (M = 0.233, SD = 0.065) and after (M = 0.221, SD = 0.038) the session (p = 0.338) and were significantly correlated (r(19) = 0.603, p = 0.008). The ‘slowest’ FMT rates clustered around 1500 ms, did not differ before (M = 1.786, SD = 0.907) and after the session (M = 1.531, SD = 0.690) and were significantly correlated (r(19) = 0.681, p = 0.001). Given the consistency of the measures, the rates for each unpaced task before and after the session were averaged for further analyses.

### Comparisons between sessions

The aim of the current study was to develop reliable measures that can estimate preferred rate and flexibility on an individual basis. To be able to assess reliability of the measures, the second experimental session was identical to the first, including the order of IOI presentation. Range of time elapsed between sessions was 5 to 20 days (M = 8, SD = 3.82). In this section, we compare results between sessions and report the reliability of the measures between repeated measurements.

We first examined the reliability of the overall performance pattern between sessions by comparing each individual’s tapping responses to the stimulus conditions that were identical between sessions. To do so, we calculated the correlation between average TME_all_ values within 30-ms IOI bins for session 1 and session 2 separately for each participant. An example participant’s bin-averages subject to the correlation analysis and all participants’ correlation coefficients are presented in Supplementary Fig. S1. One obvious question is whether the strength of this correlation was weaker for individuals with a longer elapsed time between sessions. Though we found a negative relationship between the number of days between sessions and correlation coefficients from binned TME_all_, the correlation was small and nonsignificant (Spearman’s r(19) =  − 0.259, p = 0.257). In sum, individuals performed consistently across sessions with respect to the stimulus IOI under the identical study conditions, and this was independent of the duration between testing sessions.

In order to assess the reliability of the effects reported here, we did two things. We correlated dependent measures across sessions, and we tested whether the measure changed overall between sessions using paired-samples t-tests. These two measures provide slightly different information. The t-test can inform us about whether, for example, participants improved in the second session because of practice, which is independent from whether values are correlated across sessions. In contrast to the analysis comparing individuals’ tapping performance at 30-ms IOI bins which was run separately for each individual, the analyses comparing general tapping patterns, the estimates of preferred rate and flexibility and the unpaced tapping behavior between sessions were run across participants.

#### General tapping patterns

**Figure 4:**
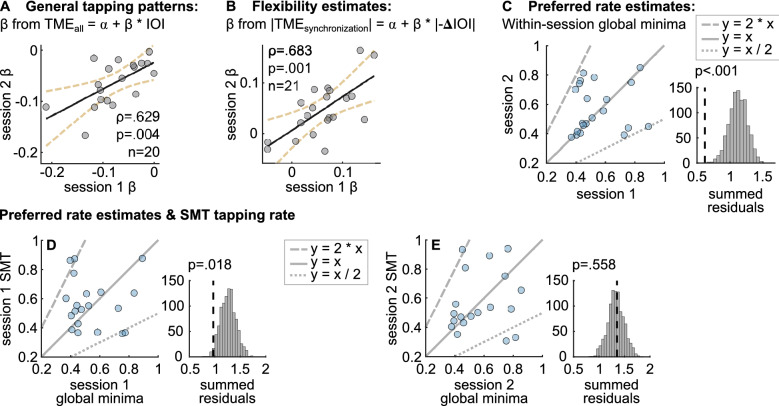
Test–retest reliability of the (**A**) IOI slopes, (**B**) flexibility and (**C**) preferred rate estimates, and (**D,E**) the relationship between preferred rate estimates from the current paradigm and SMT tapping rates. In (**A**) and (**B**), each circle represents a single participant’s β estimates from the equations TME_all_ = a + β × IOI and |TME_synchronization_|= a + β × |− ΔIOI|, respectively. In (**C**), each circle represents a participants’ within-session global minimum estimate (local minimum of the fitted curves where predicted |TME_continuation_| was minimum within a single session). Straight line represents 1:1 correspondance, and the dashed lines represent 2:1 and 1:2 ratio between x and y axes. The histogram on the right shows the permutation test results: the distribution of summed residuals (distance of the data points to the closest y = x, y = 2 × x and y = x / 2 lines) with shuffled data over 1000 iterations, and the summed residual from original data (dashed line). On the bottom panel, within-session global minima in (**D**) session 1 and (**E**) session 2 were plotted against the mean SMT tapping rate of the respective sessions. As in (**C**), the histograms show the permutation test results for each session.

In order to assess whether the effect of stimulus rate on |TME_all_| differed between sessions, we compared the IOI slopes (i.e., β estimates obtained from the TME_all_ = a + β × IOI equation) between sessions. We found a significant correlation (r(18) = 0.629, p = 0.004), and no significant difference between mean β from the first (M =  − 0.077, SD = 0.052) and second (M =  − 0.071, SD = 0.053) session (p = 0.181), showing that the asymmetry in tapping errors between the fast and slow stimulus rates was consistent across repeated measurements. The correlation of β values between sessions is presented in Fig. 4A.

#### Preferred rate measures

We inspected the test–retest reliability of our preferred rate estimates at two levels: we first compared the local minimum estimates, then the within-session global minimum estimates across sessions. Note that we estimated up-to three local minima per participant and session, and among the local minima, global minimum was the one with the smallest predicted |TME_continuation_|. We assessed between-session reliability of local minima by running a linear mixed-effects model where local minima obtained in both sessions was the dependent variable, and session was the fixed factor. The results revealed no significant effect of session (Estimate ± SE: − 0.028 ± 0.043, t =  − 0.656, p = 0.514). To assess the between-session compatibility of global minima, we developed a permutation test that accounted for the harmonic structure and the resulting bimodality of the data (see “Methods” section). Results showed a significant relationship between the two-session global minima (p < 0.001). The scatterplots and the permutation test results are shown in Fig. 4C.

#### Flexibility measures

Our final measure for flexibility was β estimates obtained from equation |TME_synchronization_|= a + β ×|− ΔIOI|, since this dependency constituted the situation that was most challenging to individuals’ oscillator flexibility. The estimates from the first (M = 0.061, SD = 0.055) and second (M = 0.047, SD = 0.053) session showed no significant difference (p = 0.166) and were correlated between sessions (r(19) = 0.683, p = 0.001), showing test–retest reliability of our measure. The correlation between the final flexibility estimates across sessions is presented in Fig. 4B.

#### Unpaced tapping measures

Finally, we compared the session-averages of tapping rates from SMT and FMT tasks. For all tasks, there were no significant differences between the mean tapping rates between sessions. Rates were also correlated between sessions. The session averages and test results are presented in Table 1.

**Table 1:** Single session descriptive statistics of measures and test results for between-session comparisons.

Measure	Session 1		Session 2		Comparison of means between sessions			Correlation between sessions		
	M	SD	M	SD	T	df	p	rho	df	p
β estimates (TME_all_ = a + β × IOI)	− 0.077	0.052	− 0.071	0.053	− 1.3929	18	0.181	0.63	17	0.004
β estimates (|TME_synchronization_| = a + β ×|− ΔIOI|)	0.061	0.055	0.047	0.053	1.438	20	0.166	0.68	19	0.001
SMT tapping rates (s.)	0.568	0.172	0.597	0.232	− 1.080	19	0.294	0.85	18	< 0.001
FMT ‘fastest’ tapping rates (s.)	0.227	0.046	0.214	0.030	1.501	17	0.152	0.63	16	0.005
FMT ‘slowest’ tapping rates (s.)	1.710	0.806	1.495	0.414	1.017	18	0.322	0.59	17	0.008

### Comparisons across measurements

#### Preferred rate and flexibility

Given the study design, the main variables stimulus rate (IOI) and rate change between trials (ΔIOI) were not necessarily fully independent, because there was a great chance that a stimulus that was fast was also presented after a slow stimulus to increase the amount of rate change, and vice versa. We aimed to choose different dependent measures that were each related to one of these two independent measures for estimating preferred rate and flexibility. However, it could still be argued that both our preferred rate and flexibility measures reflected the way individuals responded to stimulus rate only, and not to *changes* in stimulus rate. For instance, if their preferred rate was slow, they might have had trouble synchronizing with a fast rate regardless of the amount and direction of rate change between trials. We tested this possibility by assessing the relationship between individuals’ preferred rate and flexibility estimates. However, since there were up to 3 preferred rate (local minima) estimates for each individual and a single estimate for flexibility in each session, we took ‘across-session global minimum’ (see Fig. 2B) as an overall measure representing individuals’ preferred rate. Finally, we averaged flexibility estimates between sessions. We compared the flexibility estimates between individuals with fast and slow preferred rate estimates by conducting a median-split on across-session global minimum distribution. Paired-samples t-test between the average flexibility estimates of individuals with fast (smaller than median global minimum) and slow preferred rate estimates revealed no significant difference between the groups (p = 0.395).

#### Preferred rate and unpaced tapping rate

In order to assess whether our preferred rate measures were compatible with other measures of rate preferences, we compared preferred rate estimates with unpaced tapping rates (SMT) in individual sessions. We used the permutation test we developed to compare two harmonically-related distributions of data, described in “Methods” section. The test revealed a significant relationship between global minima and average SMT in the first session (p = 0.018), but not in the second session (p = 0.558). The relationship between the preferred rate estimates and the SMT tapping rates and the distributions of summed residuals from the permutation test for session 1 and session 2 are presented in Fig. 4D,E, respectively.

## Discussion

In the current study, we assessed two properties of internal oscillators that we assume to underly entrainment to auditory rhythms: their preferred rate and flexibility. Here, we defined preferred rate as the stimulus rate(s) that a person could tap to with minimum error, based on the assumption that these rates reflect the natural frequency of individuals’ internal oscillators that is at work in the absence of an auditory signal. We conceptualized flexibility as the ability to adapt to changes in external auditory context. In order to estimate preferred rate and flexibility for each individual, we ran a synchronization-continuation tapping experiment where individuals synchronized with a 5-event isochronous stimulus sequence and continued the rhythm at the same pace. The dependent measure was tempo-matching error, calculated as the proportional rate differences between stimulus and tapping rate. All participants completed two identical sessions so that we could assess the reliability of the measures derived from our novel paradigm.

**Figure 5:**
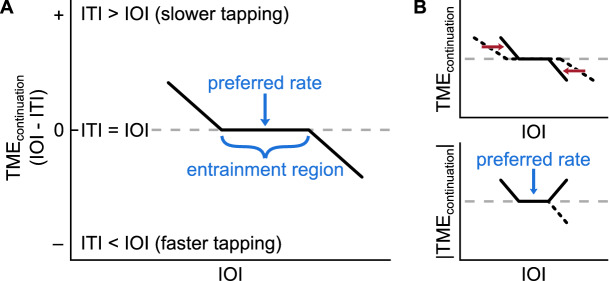
(**A**) “A theoretical detuning function” for an entraining oscillator, its preferred rate and the entrainment region. Adapted from Ref.^6^. At fast rates outside of the entrainment region, TME_continuation_ is positive since tapping intervals are longer than the stimulus intervals due to drift to the preferred rate. At slow rates, the opposite is true. (**B**) An illustration of the hypothesized contraction of the entrainment region due to the ΔIOI manipulation. The lower plot illustrates how preferred rate was captured by our estimation method, curve fitting to the absolute values of TME_continuation_; and that the contraction would leave preferred rate unaffected.

Based on the dynamic attending framework, and specifically, the preferred period hypothesis^6^, we predicted a negative relationship between tempo-matching errors (TME) and stimulus IOI, which we referred to as IOI slope, meaning that individuals would tap too slow in response to fast rhythms and vice versa. As expected, the overall tapping patterns revealed a negative IOI slope for most individuals**.** Rate^17^ and interval^29^ reproduction studies, as well as synchronization-continuation paradigms^6,7^ reveal similar results, in that produced intervals in reproduction or continuation tapping tend to be (relatively) slower than the stimulus at fast rates, and faster than the stimulus at slow rates. Both interval and oscillator models are capable of explaining the presence of negative IOI slope. From an interval perspective, the negative relationship between reproduced rates and stimulus rate has been attributed^17,29^ to the draw of reproduced rates towards the overall mean of the presented rates—the ‘central tendency effect’^17^. Critically, this account presupposes ‘priors’ for interval or rate representations based on some averaging of the rates presented over the course of the experiment. From an oscillator perspective, negative IOI slope is consistent with the existence of an ‘entrainment region’^6^: an individual’s internal oscillator is capable of entraining within a range of rates around their preferred period^30^. Outside of the entrainment region, the oscillator lags at fast rates and leads at slow rates. The entrainment region is hypothesized to start relatively narrow in early stages of life, expand in adulthood, and contract again with old age. An illustration of the theoretical entrainment region is shown in Fig. 5A. In the current study, we should also acknowledge that one contributor to IOI slope might be motor constraints^31^ which would introduce positive TME_all_ (slower tapping) at fast rates, compatible with similar findings of increased tapping intervals in fast (IOI = 200 ms) conditions^7^. In fact, the average ‘fastest’ forced motor tempo (FMT) tapping rates our participants produced were actually slower than the fastest stimulus conditions in our study, suggesting that the fastest stimulus rates may have been uncomfortable to synchronize with and this may have contributed to positive TME_all_ at fast rates.

The preferred period hypothesis^6^ predicts that individuals’ productions will drift towards their preferred rates during continuation tapping. On that basis, we hypothesized that individuals’ preferred rates would be identifiable as reduced absolute normalized tempo-matching errors specifically in the continuation section of the trials (|TME_continuation_|). Conversely, we expected relatively high |TME_continuation_| at rates further from individuals’ preferred rates due to a greater degree of drift at these rates back towards preferred rate. Finally, we expected a correspondence between individuals’ preferred rate estimates based on |TME_continuation_| and their SMT. As expected, |TME_continuation_| fluctuated over the IOI range, and we identified estimates of preferred rate where |TME_continuation_| was minimal. Although we allowed for up to 3 estimated local minima for each individual’s single session data, estimates from all participants formed a bimodal distribution with peaks around 400 ms and 800 ms. Previous studies reveal similar results in terms of the range and the bimodality of individuals’ preferred rates^7,9,32^. However, they measure preferred rate by means of SMT and report the distributions across all participants, in contrast to the individual-based estimates here. To investigate the source of bimodality here, we assessed the possibility of harmonic structure of the individual estimates. We normalized the local minimum estimates of each participant by their fastest local minimum estimate both within and across sessions. The means of the normalized local minima distributions did not differ from 2 in individual sessions, and the two-session normalized estimates had a bimodal distribution with peaks around 1 and 2, suggesting test–retest reliability and harmonic structure of the estimates, respectively. Further investigation of continuation tapping within individual trials revealed that participants did indeed drift towards their preferred rates (rather than consistently overproducing and underproducing fast and slow rates, respectively, without drifting), but only on trials where stimulus rate was slower than their preferred rate. Finally, we observed one-to-one correspondence and harmonic relations between individuals’ preferred rates and their SMT: for some participants, SMT was close to the double or half of preferred rate estimate (see Fig. 4D,E). However, the permutation test we ran to quantify the systematic relationship between preferred rate estimates (as indexed by global minimum, the stimulus rate with minimum predicted |TME_continuation_|) and their SMT tapping rate was significant only for the first session.

Our work here on preferred rate supports and adds to the existing literature in several respects. First, in contrast to the SMT task which is commonly used to assess individuals’ preferred rate, our novel paradigm enables assessment of characteristics of rhythmic entrainment under a performance rather than a preference context. This distinction renders our method potentially more reflective of internal oscillator properties than the tapping rate in an SMT task, given poor evidence in the literature empirically relating SMT to the underlying oscillatory processes. Other performance-based paradigms^6,7^ also reveal reduced error in continuation tapping, centered around individuals’ SMT. However, these paradigms rather assess tapping behavior in a limited number of stimulus conditions with respect to individuals’ SMT. In contrast, with the aim of preferred rate estimation, we presented individuals with finely sampled stimulus rates, which enabled precise estimates of preferred rate(s). Moreover, stimulus rate ranged from 200 ms (5 Hz) to 1000 ms (1 Hz) which gave adequate space for the estimates. With the combination of these properties, we were able to observe multiple rates at which tapping error was minimal (harmonics). In a paradigm such as SMT where individuals produce a single tapping rate, such a finding would be implausible. One possible explanation for the potentially weaker-than-expected relationship between the preferred rate estimates and the SMT tapping rate is the differences between the tasks with the latter involving no prior entrainment to any stimulus.

The findings regarding individuals’ preferred rate support the entrainment account in a critical way: the harmonically related local minimum estimates support the hypothesis that multiple nested oscillators might be at play in rhythmic entrainment^33^. The ability to respond to multiple stimulus rates within a temporal hierarchy is a critical property of oscillatory systems^34,35^, which is not predicted by interval-based accounts of time perception^20^. Moreover, harmonic relations between the estimates are not compatible with the ‘central tendency’ interpretation of the asymmetry between tapping errors at fast and slow rates, given the singular nature of the ‘priors’ for rate representations. Finally, oscillator models and the entrainment approach predict that when oscillator that is perturbed away from its natural frequency will gradually return to its natural frequency after the perturbation^2,22,35^. In the context of continuation tapping, this tendency would result in drift towards the preferred rate of the oscillator. Compatible with the previous findings from tapping paradigms^11,12^, we found drift towards preferred rate in the current study. However, we did not observe slowing-down in continuation tapping at rates faster than preferred rate, which might be due to the asymmetry between the number of trials where stimulus was faster or slower than individuals’ preferred rates, given that preferred rate estimates for most individuals clustered around 400 ms, faster than the mean of all rates that were presented. It can also be that the allowed duration to produce continuation taps was too short for a robust analysis for drift.

We challenged individuals’ flexibility by maximizing the rate differences (ΔIOI) between consecutive trials. Based on the evidence for local and global context effects on timing responses^36^, in addition to the hypothesized ability of internal oscillators to adapt to external rhythms^20^, we predicted that absolute normalized tempo-matching errors at the synchronization section of the trials (|TME_synchronization_|) would be increased with increasing ΔIOI, especially in the more challenging conditions where stimulus rate was faster than previous. As predicted, |TME_synchronization_| increased with ΔIOI, but only when it was negative (in conditions where stimulus rate was faster than the one in the preceding trial). In contrast, |TME_synchronization_| decreased with positive ΔIOI, showing that slower-than-previous stimuli were easier to synchronize to. Having conceptualized flexibility as robustness to effects of stimulus history, we chose β estimates obtained from the model where |TME_synchronization_| was predicted by |− ΔIOI| for our final flexibility measure, with a higher value indicating more inflexibility.

It is a common finding across a number of domains that the history of stimuli an individual has heard or seen influences their perception of (or at least responses to) the current stimulus. For example, these so-called “history effects” are present in orientation discrimination^37^ and face identification^15,16^ in vision, and in pitch judgements^18,26^ and rate reproduction^17^ in audition. The strength of these history effects is modulated by factors such as sensory uncertainty^15^, attention, and temporal proximity between the stimuli^37^ in vision and response inhibition^17^ and global temporal context^36^ in audition. In the current study, high trial-to-trial stimulus uncertainty and the fast pace of stimulus presentation unsurprisingly led to robust history effects. Moreover, the magnitude and direction of the history effects differed depending on the stimulus rate of the preceding context: tapping errors increased with faster trials and decreased with slower trials. Studies investigating temporal adaptation reveal similar findings: synchronization with sequences that are slowing down result in smaller asynchronies between the taps and event onsets than sequences that are speeding up^22–24^. Current findings thus complement previous research by showing the asymmetry also in tempo-matching errors, which highlights the role of period correction in temporal adaptation. This asymmetry may result either from motor constraints that would be more pronounced for fast tapping, or from perceptual phenomena. In fact, studies have shown better detection of time-change for late targets than for early targets^2^, and higher sensitivity for detection of positive deviations (late onsets) than for negative deviations^38^ in rhythm perturbation paradigms, and better accuracy for detection of late offsets than for early offsets in a rhythm extrapolation/timekeeping task^39^. At the theoretical level, the interval model predicts no difference in synchronization errors to either direction of rate change, whereas the oscillator model predicts increased errors to speeding up than to slowing down sequences due to the nonlinearity of the period adaptation function of the oscillator^23^. Thus, our findings are more compatible with the latter model and the entrainment account.

We chose to focus on data from different parts of each trial in our analyses of the effects of IOI and ΔIOI, that is, |TME_synchronization_| and |TME_continuation_|, respectively. Since our analysis pipelines related to the two effects are also independent, we have treated our discussion of preferred rate and flexibility separately. However, we recognize that it could be argued that both sets of results arose from the existence of a preferred IOI at which TME was minimal. For instance, if an individual’s preferred rate was slow, synchronizing to a faster rate would have been more challenging for them, and vice versa. Thus, our history effects may have been a by-product of difficulty synchronizing at rates far from one’s preference, as opposed to difficulty flexibly updating an internal oscillator’s period. However, we found no relationship between individuals’ preferred rate and flexibility estimates, which we interpret to mean that the possibility that individuals were responding only to stimulus rate based on their preferred rate was unlikely.

Based on our experience with this paradigm and data, we have started to develop a hypothesis regarding the interplay between preferred rate and flexibility in the context of the entrainment region hypothesis. We suspect that our fast-paced design with high stimulus uncertainty might have contracted individuals’ entrainment region, which is defined as the range of rates around the preferred rate to which an individual can successfully entrain (Fig. 5A; Ref.^6^). In that case, individuals would still entrain best to their preferred rates, but the increase in tapping errors around that rate might be influenced by the ΔIOI manipulation. Figure 5B illustrates the hypothesized contraction of the entrainment region, our method for preferred rate estimation, and the robustness of preferred rate to the contraction. In a less challenging synchronization-continuation paradigm that did not involve a rate-change manipulation, within the IOI range of the current study, McAuley et. al. have found that the entrainment region was wide for young adults (18–59 years), and narrow for children (4–12 years) and older age groups (60+)^6^. In the current study, tapping errors that are stable across the stimulus range would render estimation of oscillator properties difficult.

One of the goals of the current study was to develop a reliable method for estimating preferred rate and flexibility. Reliability is a crucial feature of any study that aims to establish a technique for measurement of cognitive phenomena^40^. In order to assess whether our methods reliably estimate preferred rate and flexibility, we ran two identical sessions of the experiment for each individual. In line with the previous findings^41^, preferred rate estimates were consistent across sessions. We found a strong between-session correspondence also for flexibility estimates and no effect of session on the estimates of either preferred rate or flexibility. Moreover, general tapping patterns across the stimulus rates (IOI slope) were also reliable across sessions. These findings show that the methods we developed produced replicable results under identical conditions. This is also important for the future use of the methods for purposes such as fine-tuning stimulus conditions to improve individuals’ rhythmic entrainment and attentional skills in the auditory domain.

Our method differed in a number of ways from previous synchronization-continuation paradigms. The most prevalent deviation was short trials (5 synchronization + 7 continuation taps). We implemented this feature in the design since we were interested in how synchronization fails, rather than how it is achieved. Low number of taps for synchronization enabled us to push individuals’ flexibility to its limits by preventing them from settling on any stimulus rate. Another feature of the design was lack of replication of identical stimulus conditions; that is, every stimulus rate was presented only once, and we finely sampled a large number of IOI conditions, which served our purpose of estimating preferred rate on an individual basis. A small number of rate conditions repeated over the course of many long trials, the approach used in previous studies^6,7,27,42^, tends to reinforce each stimulus rate because of a lack of discrepancy between the rates in individual trials, as well as between local and global IOI. We aimed to avoid such reinforcement, which enabled individuals’ preferred rates to pervade independent of any “priors” the participants may have formed based on repetitions of local and global context rates^29^.

With the goal of reliably estimating individuals’ internal oscillator’s preferred rate and flexibility properties, we conducted a synchronization-continuation tapping experiment. We developed methods to measure preferred rate and flexibility based on how individuals responded to different stimulus rates, as well as to the challenge of abrupt rate changes. The results not only revealed reliable estimates across sessions, but also supported ‘the entrainment approach’ to rhythm perception, on which we based the assumptions to build our design in the first place. The current findings constitute an important step in characterization of oscillator properties and demonstrate the influence of properties of the auditory context on rhythmic entrainment, which may shed light onto how auditory and attentional synchronization can be improved in future research.

## Methods

### Participants

23 participants (mean age: M = 27.19, SD = 6.38) were recruited from Frankfurt am Main and surrounding areas in Hessen, Germany. All participants were native German speakers and self reported normal hearing. All participants were drawn from the participant pool of the recruitment system of Max Planck Institute for Empirical Aesthetics laboratories. They received 21 Euros per session for compensation. Written informed consent was obtained from all participants. The procedure was approved by the Ethics Council of the Max Planck Society and was in accordance with the Declaration of Helsinki. Prior to the experimental sessions, participants completed an online background survey. Two participants completed this survey after the sessions. Participants’ demographic information is summarized in Supplementary Table S1.

### Apparatus

Stimuli were generated and presented on a Windows desktop computer, using the Psychophysics Toolbox extensions^43,44^ for Matlab. Auditory stimuli were presented via Beyerdynamics 880 pro headphones. Each participant’s hearing-level threshold for a white-noise stimulus was first determined using the method of limits following the procedure described in Ref.^45^. Then, the sound level for experimental stimuli was adjusted to + 55 dB above each participant’s hearing threshold individually for each session (sensation level^45^). All instructions were presented on a ASUS VG24QE LCD screen. Tapping responses were collected via a Schaller Oyster S/P contact microphone, attached on the right half of the desk, at a sampling rate of 44,100 Hz. Participants were instructed to tap one finger on the table next to the microphone with the same strength throughout the session. Audio was presented and recorded by RME Fireface UC soundcard.

### Stimuli

Stimuli were sequences comprising 5 woodblock sounds. Woodblock samples (originally 213 ms) were cut to 100 ms in duration and faded out with a linear ramp to smooth out the end. Note that the quality of the woodblock sound was not changed by shortening it. A single trial consisted of an isochronous stimulus sequence followed by silence. The inter-onset-interval (IOI) of the woodblock sounds making up each trial’s stimulus sequence varied between 200 and 1000 ms in 2-ms steps. For example, stimulus IOI was 200 ms in one trial, and 202 ms in another trial, and so on. Each stimulus having a different IOI resulted in 401 different stimulus rates being presented on 401 different trials.

### Procedure

All participants completed a series of motor timing tasks. An unpaced motor task consisted of a ‘spontaneous’ motor tempo (SMT) measurement, which is commonly used to assess preferred rate^3,6,7^, and a ‘forced’ motor tempo (FMT) task where participants tapped at the fastest and slowest rates that they were able to, for assessment of the range of free tapping rates within their motor abilities. The main task was a synchronize-continue tapping task performed at a wide range of stimulus rates. The main tapping task also required rapid adaptation to changes in stimulus rate from trial to trial. In all motor tasks, participants tapped on a table with their dominant hand, using their index finger, as instructed. Details of all motor tasks are provided below.

All participants completed two identical sessions, separated by 5–20 days. A single session started with the sound-level threshold test, followed by the unpaced SMT and FMT tasks, respectively. Then, participants were presented with instruction text and figures on the computer screen that explained the main task. A practice block simulating the main task followed the instructions (details below). The main section consisting of 401 trials started after the participants agreed that they understood the task. A short, optional break was offered midway through the main task, after 200 trials. Lastly, unpaced tapping tasks were repeated in the same order. Participants were debriefed upon their request, only after the second session. An individual session lasted 80 min on average. The experiment procedure is illustrated in Fig. 1.

#### Unpaced tapping tasks

In the spontaneous motor tempo (SMT) task, participants were asked to “tap on the desk at a rate that is comfortable to maintain”. In the ‘forced’ motor tempo (FMT) tasks, participants were asked to “tap at the slowest rate that is comfortable to maintain” and “tap at the fastest rate that is comfortable to maintain”. The duration allowed for the tapping responses was 30 s in the SMT task and ‘fastest’ FMT task, and 45 s in the ‘slowest’ FMT task. The unpaced tasks were run twice in each session in the same order, where the SMT task was followed by the ‘slowest’ FMT and then by the ‘fastest’ FMT task.

#### Synchronize–continue tapping

Participants were instructed to “start tapping along with the sounds as soon as the sounds start and keep tapping at the same pace once the sounds stop, until the screen changes color”. They synchronized with the isochronous stimulus for 5 taps (4 intervals); then, the sounds stopped, and they continued tapping at the same rate for another 6 or 7 intervals. Allowed duration for continuation tapping was 7 intervals for stimulus IOI smaller than 300 ms, and 6 intervals for those bigger than 300 ms, in order to control for the total tapping duration. The end of each trial was signaled by a change in screen color from white to blue. Duration of the inter-trial interval, during which the screen remained blue, was randomly selected from a normal distribution with a mean of 1000 ms (SD = 50 ms).

The inter-onset-intervals (IOIs) separating the woodblock sounds making up each stimulus sequence were fixed within a trial, but across trials were linearly spaced between 200 and 1000 ms in 2-ms steps, resulting in 401 different stimulus rates. Every stimulus IOI condition was presented once in each session. Differences in stimulus rate between trials (referred to here as ΔIOI) were maximized from one trial to the next, and the direction of the change (i.e., whether a trial was faster or slower than the previous) alternated on every trial. That is, if the stimulus IOI on one trial was faster than the previous, the following trial would be slower, and vice versa. It should be noted that the stimulus in a single trial was always isochronous, and the IOI changed only between trials. The event structure of two consecutive trials is illustrated in Fig. 1.

Prior to the main task, participants were trained in a practice block consisting of 6 trials. After each practice trial, a feedback text appeared on the screen, which informed them if they tapped too fast, tapped too slow, started tapping too late, or finished tapping too early. Two practice trials each were presented at slow, medium, and fast IOIs; stimulus IOIs for practice trials were randomly selected from ranges of 734–1000 ms, 467–733 ms and 200–466 ms, respectively.

#### Background survey

In order to capture information about factors that might have a role in tapping performance, such as age and musicality, each participant completed an online test battery. The first part of the survey consisted of questions about participants’ demographics, language skills, hearing abilities, and psychological disorders. The second part was ‘The Goldsmiths Musical Sophistication Index’^46^. All questions were in German.

### Design

The independent variables were stimulus IOI and difference in stimulus IOI between a trial and the previous trial, ΔIOI. ΔIOI could be positive or negative, indicating that the stimulus on a trial was slower or faster than the one in the previous trial, respectively. For example, if stimulus IOI was 600 ms in trial_n_ and 550 ms in trial_n-1_, ΔIOI in trial_n_ was + 50 ms.

The primary dependent variable was *tempo-matching error (TME)*. We were interested in how inaccurately each individual reproduced the single-trial stimulus IOI, as a function of stimulus IOI and as a function of ΔIOI. Thus, for each trial, we subtracted stimulus IOI from tapping rate (median inter-tap-interval), and normalized this difference by stimulus IOI, as shown in Eq. (1).

1 $${\mathrm{TME}}_{k}=\frac{ \left(\mathrm{median }[{\mathrm{ITI}}_{1}, {\mathrm{ITI}}_{2}, \dots , {\mathrm{ITI}}_{\mathrm{n}}]\right)-{IOI}_{k}}{{IOI}_{k}}$$

where k is the trial index and n is the maximum number of intervals in a single trial.

Thus, TME is a directional, proportional error measure. A positive TME indicates that the participant tapped slower than the stimulus IOI, and a negative TME indicates that the participant tapped faster than the stimulus IOI.

In order to test the hypothesis of a negative slope for tempo-matching errors as a function of stimulus rate, TME was calculated from both synchronization and continuation parts of the trial (i.e., from all taps). Independent variable for this hypothesis was stimulus IOI and the dependent variable was TME_all_. To test the second hypothesis of increased tempo-matching errors during continuation of the stimuli, especially at rates that were far from the individual’s preferred rate, we focused on continuation tapping. Independent variable was stimulus IOI, and the dependent variable was |TME_continuation_| since we were interested in tapping accuracy regardless of its direction. In the analyses testing the hypothesis that trial-to-trial rate changes would result in increased tempo-matching errors during synchronization, independent variables were absolute values of − ΔIOI and + ΔIOI, (how much faster or slower a trial was from the preceding trial), and the dependent variable was absolute values of TME, calculated from the synchronization part of the trials (|TME_synchronization_|).

For all unpaced tapping tasks, the dependent measure was the produced rate, quantified as the median of all ITIs in a single trial after data cleaning.

### Analysis

#### Preprocessing and data preparation

Tapping data were collected by a microphone, thus individual tap times had to be isolated in the continuous audio recording. We developed a procedure that enabled flexible thresholding for extraction of the taps from the sound recordings. For each trial’s recording, the script first extracted all amplitudes that exceeded the manually specified noise floor present in the recording. Then, a second, more conservative threshold was applied that retained all peaks whose amplitude exceeded 30% of the median peak amplitude value; that is, the top 70% of identified amplitude peaks were retained as “taps”. We arrived at this procedure by trial-and-error, and found that it enabled extraction of low-amplitude taps, which would fall under the threshold and be omitted if a fixed noise threshold was used. Finally, an automated procedure detected taps that marked intervals that were smaller than half, or larger than 1.8 times the stimulus IOI, plotted the recording for visual inspection, and these trials were manually cleaned from sound artifacts that were falsely detected as taps.

Inter-tap-intervals (ITI) were calculated as the difference between neighboring taps’ timestamps. For each trial, synchronization ITIs referred to all intervals produced before the end of the sound stimulus, while continuation ITIs occurred after the sound stimulus ended.

#### Data cleaning and exclusion criteria

Single-trial ITI outliers were detected and removed using an automated procedure. In a first step, intervals were marked whose deviation from the median ITI exceeded 3× the median absolute deviation (MAD) of all ITIs in one trial^47^. In a second step, a linear regression was fitted to all unmarked ITIs as a function of time within that trial. Marked ITIs were compared to the value predicted by the regression model, and any ITI that was smaller than half or larger than 1.5 times the predicted ITI was removed. We adopted this outlier-detection method based on linear regression to account for ITI drift that may have occurred within trials. The same procedure was followed in the analysis of unpaced tapping tasks as well.

Any trial that had less than 5 taps (4 ITIs) after the end of the sound stimulus, i.e., less than 4 continuation ITIs, was removed. Finally, every trial’s median ITI and the corresponding stimulus IOI information were submitted to hierarchical clustering analysis with a single distance criterion, and any ITI that did not fall into the main cluster was removed. This method constituted a standardized exclusion criterion while allowing different IOI-ITI relationships for different participants.

After these exclusions, one participant was removed from the analysis because we were able to retain less than 80% of all trials in both sessions. We also adopted an exclusion criterion at the participant level that required participants to have similar tapping patterns across sessions (over all IOIs). In order to test this, we compared participants’ tapping rates between sessions. However, comparison of identical conditions across sessions was difficult because the study design had no replications of individual trials and data cleaning procedures at the trial level omitted different trials for individual sessions. Thus, we first binned the TME_all_ data into 30-ms stimulus-IOI bins and averaged within each bin. Then, for each participant, we assessed correlation between the mean TME_all_ values of 26 bins. One participant was removed from further analysis because of a negative correlation in tapping errors across sessions (see “Methods” section for correlation coefficients for the remaining participants).

For data cleaning of unpaced tapping tasks, every trial’s median ITI was calculated first. For each trial, the distribution of resulting tapping rates across participants was inspected separately for each unpaced task, and any participant that exceeded 3× the median absolute deviation (MAD) of all tapping rates were excluded from further analysis.

#### General tapping patterns

In order to assess how individuals responded to the stimulus rate, we quantified the effect of stimulus rate on tempo-matching errors of all taps (TME_all_). To do so, we fitted linear models (TME_all_ = α + β × IOI) to individual-participant, single-session data, and retained the β estimates obtained from these models. Then, we excluded any participant that exceeded 3× the median absolute deviation (MAD) of all β estimates from further analysis.

#### Preferred rate measures

In order to assess how tempo-matching errors during continuation tapping varied across the IOI range, we examined the relationship between stimulus IOI and |TME_continuation_|. However, in contrast to the other analyses reported here, we predicted a nonlinear relationship between the variables, based on two predictions of the entrainment approach: increase in tapping errors as the difference between the oscillator and stimulus rate increases^4,6^ and better entrainment to stimulus rates that are harmonically related to preferred rate^33^—or at different ‘metrical levels’^22^.

For each individual and each session, we used absolute values of TME_continuation_, |TME_continuation_|, to quantify preferred rate because we were interested in identifying the stimulus IOIs that were associated with the smallest tapping errors (best tempo-matching) regardless of the direction of the error (whether taps were faster or slower than the stimulus). Given the design of the current study that involved a large number of different stimulus rates and no repetition of a stimulus condition, averaging across stimulus conditions was not possible. In order to estimate the rates at which individuals had the smallest |TME_continuation_| values from these noisy, single-trial data, we developed a curve fitting analysis. Curve fitting involves fitting a function to a set of data points, and is useful for reducing the data to a manageable form and investigating the relationships between variables^48^. As the function to fit to the data, we chose sum-of-sines, since it would capture the fluctuations in the data and allow for estimation of multiple, harmonically related ranges where |TME_continuation_| was minimal. We fitted a sum-of-sines equation to |TME_continuation_| values as a function of stimulus IOI, allowing for a maximum of 7 sine components and three minima. We limited the number of minima to three, because this is the maximum number of harmonically related troughs that could occur in the stimulus IOI range of 200–1000 ms. The final estimates of preferred rate were the local minima of these fitted curves, indexing the best-performance rates across the stimulus rates. Each participant had between 2 to 4 sine components and between 1 and 3 minima, and most participants had 2 minima (M = 1.64, SD = 0.62). An illustration of the curve fitting procedure to an example participant’s data is provided in Fig. 2B.

We assessed test–retest reliability of the preferred rate measures at multiple levels. First level was the local minima. Since the estimates consisted of multiple values obtained for each participant, assessment of between-session comparisons using correlation methods was not appropriate. Thus, to assess whether session was a significant predictor of local minimum estimates, we ran a linear mixed-effects models (LMEM) analysis, using the fitlme function implemented in MATLAB. Dependent variable was all local minimum values obtained on an individual basis, and session was the fixed factor.

On the second level, we compared individuals’ global minimum estimates (minimum predicted |TME_continuation_| values on the fitted curves) across sessions. However, for some participants, global minimum obtained in the first session was close to half or double of the value obtained in the second session, suggesting harmonic structure, as illustrated in Fig. 4C where some data points fall around the y = 2 × x and y = x / 2 lines. For assessment of between-session compatibility of global minima, we developed a permutation test that accounted for the harmonic structure, and the resulting bimodality of the data that rendered the correlation methods unusable. First, we calculated the absolute distance between predicted and observed value, namely, the residuals^49^ of each data point. In linear regression, the relationship between the predictor and outcome variable is determined by fitting a line that gives the least amount of (mean) residuals^49^ in the data. Here, the residuals correspond to the perpendicular distance between the data points to the closest line among the y = x, y = 2 × x and y = x / 2 theoretical lines, representing 1:1, 2:1 and 1:2 ratio between the variables (for comparison of distance methods, see Ref.^50^). The resulting measure was the sum of all residuals. Finally, we shuffled the Y-axis values (second session global minima) with respect to the X-axis values (first session global minima) 1000 times and obtained the summed residuals for each permutation. The test statistic was the percentage of summed residuals that fell under the initial value that was computed from original data points.

To test whether participants drifted towards their preferred rate during continuation tapping, we examined how tapping intervals changed within each trial. To do so, we fitted linear models to each trial’s continuation tapping, where ITI was predicted by tap number (ITI = α + β × tap number), and obtained slopes (β). A positive slope indicates an increase in continuation ITI over each tap, namely, a slowing in the tapping rate. A negative slope indicates that the tapping during continuation got faster. Next, we separated slopes into two groups: slopes obtained for the trials with stimulus rates that were faster than an individual’s preferred rate estimate (within-session global minimum), and those on the trials that were slower. For each participant and session, we averaged slopes within the two groups. Finally, for each session, we ran separate one-sample, one-tailed t-tests to assess whether participants’ mean slopes were significantly larger or smaller than zero. We also compared the mean slopes across sessions for both groups. Before the tests, we removed an outlier value that exceeded 1.5 interquartile range above the upper quartile of session 1 averages of slower-than-preferred rate trials’ slopes.

#### Flexibility measures

In order to assess adaptation to rapid rate changes, which we conceptualized as a challenge to oscillator flexibility, we quantified the effect of ΔIOI on tempo-matching errors from the synchronization section of the trials, namely, TME_synchronization_. Since we were interested in the strength of the rate-change (ΔIOI) effect on TME_synchronization_ regardless of the direction of the error, we took absolute values of TME_synchronization_. We assessed the effects of both positive and negative ΔIOI on |TME_synchronization_|, and took absolute values of ΔIOI for comparisons. In order to quantify the effect of |ΔIOI| on |TME_synchronization_|, we fitted linear models (|TME_synchronization_| = α + β ×|− ΔIOI| and |TME_synchronization_| = α + β ×|+ ΔIOI|) to individual-participant, single-session data, and retained the β estimates obtained from these models. The analysis used to estimate flexibility is illustrated in Fig. 1.

## Supplementary Information

Supplementary Information.

## Data Availability

The datasets collected and analysed during the current study are available from the corresponding author on request.
