# Supplementary material for: Reliable estimation of internal oscillator properties from a novel, fast-paced tapping paradigm
Source: Sci Rep. 2022 Nov 28;12:20466. doi: 10.1038/s41598-022-24453-6 (PMC9705557; doi:10.1038/s41598-022-24453-6)
Supplement: Supplementary file 1 — Supplementary Information. [file 41598_2022_24453_MOESM1_ESM.pdf]

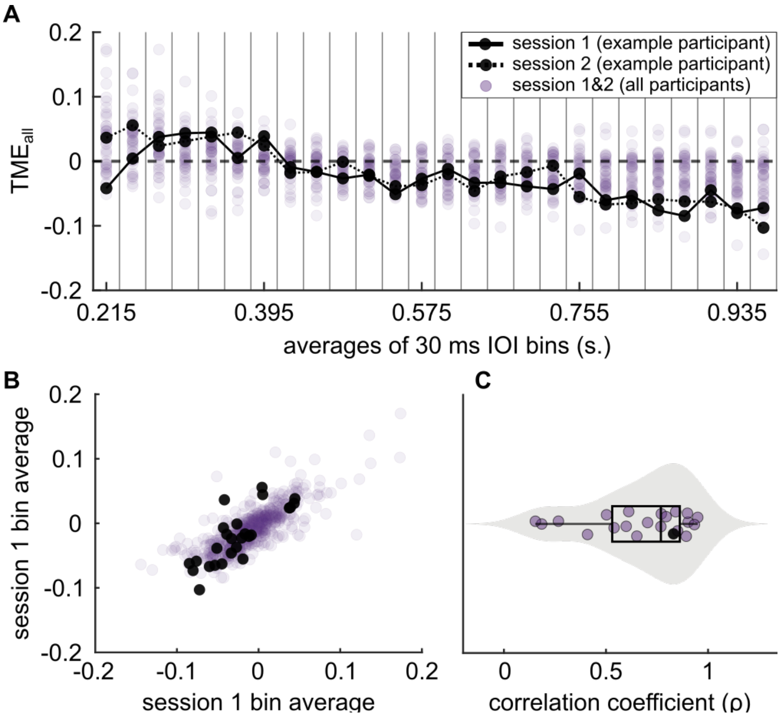

Supplementary Figure S1. (**A**) Average TME_{all} values of 30-ms IOI bins. Shaded circles represent individual participants. Black circles and the connecting lines represent an example participant's bin averages in first (straight line) and second (dashed line) sessions. Vertical black lines show bin edges. (**B**) Between-session scatterplot for average TME_{all} values of 30-ms IOI bins. Shaded circles represent individual participants. Black circles represent the example participant's data, shown in black in panel A. (**C**) The distribution of correlation coefficients, obtained for 21 participants. Box plots show median (black vertical line), 25th and 75th percentiles (box edges) and extreme datapoints (whiskers). Each circle represents a single participant. Black circle represents the example participant's coefficient.

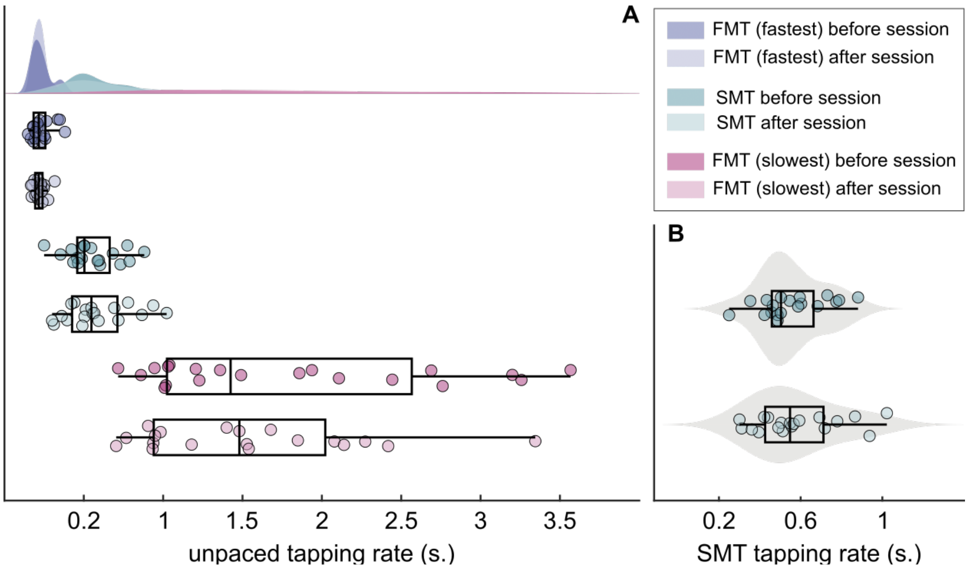

Supplementary Figure S2. (**A**) Unpaced tapping rates from SMT (teal) and FMT tasks with 'fastest' (purple) and 'slowest' (pink) tapping in session 1. (**B**) SMT tapping rates in session 1 on a bigger scale. Dark and light shades for each color represent tapping rates measured before and after session, respectively. Box plots show median (black vertical line), 25th and 75th percentiles (box edges) and extreme datapoints (whiskers). Each circle represents a single participant.

Demographic variables	N	Percent	Min	Median	Max
Age	23	100	21	25	51
Gold-MSI General Musical Sophistication	23	100	34	66	97
Gender	23	100			
Female	17	74			
Male	6	26			

Survey responses	N	Percent
Which hand do you use for writing?	21	91
Left	1	4
Right	20	87
Which is your highest professional training qualification?	22	96
I have not finished any career/job training and I am not within such a training	1	4
I am still in my career/job training/intern/student	10	43
Bachelor on university (of applied sciences)	5	22
Graduated from a university of applied sciences (Diplom, Master); graduated from a university (Diplom, Magister, Staatsexamen, Master)	6	26
Native Language	22	96
German	17	74
Bilingual	5	22
German, Arabic	1	
German, Chinese	1	
German, Polish	1	
German, Vietnamese	1	
German, Hungarian	1	
To which of the following music genres are you listening to most often?	23	100
Rock	6	26
Pop	6	26
Classical music	2	9
Jazz	1	4
Metal	2	9
Electronic dance music	3	13
Rap	1	4
Hip-hop	1	4
Music from other cultures	1	4

Supplementary Table S1. Participants' (N=23) demographic information and survey responses. The Goldsmiths Musical Sophistication Index (Gold-MSI) General Musical Sophistication scale upper and lower score limits are 126 and 18 points, respectively.
